# Effects of Chronic and Experimental Acute Masseter Pain on Precision Biting Behavior in Humans

**DOI:** 10.3389/fphys.2019.01369

**Published:** 2019-10-29

**Authors:** Samaa Al Sayegh, Annie Borgwardt, Krister G. Svensson, Abhishek Kumar, Anastasios Grigoriadis, Nikolaos Christidis

**Affiliations:** ^1^Division of Oral Diagnostics and Rehabilitation, Department of Dental Medicine, Karolinska Institutet, Huddinge, Sweden; ^2^Scandinavian Center for Orofacial Neurosciences, Huddinge, Sweden; ^3^Swedish Armed Forces Headquarters, Medical Service, Stockholm, Sweden

**Keywords:** jaw motor control, chronic pain, myalgia, experimental pain, hypertonic saline

## Abstract

Chronic pain in the orofacial region is common worldwide. Pain seems to affect the jaw motor control. Hence, temporomandibular disorders (TMD) are often accompanied by pain upon chewing, restricted mouth opening and impaired maximal bite forces. However, little is known on the effects of pain, in particular the effects of chronic jaw muscle pain on precision biting. The aim of the study was to investigate the effect of chronic and acute jaw muscle pain on oral motor control during precision biting in humans. Eighteen patients with chronic masseter muscle pain and 18 healthy participants completed the experiment. All participants were examined according to the Diagnostic Criteria for TMD. Experimental acute pain was induced by bilateral, simultaneous sterile hypertonic saline infusions into the healthy masseter muscles. A standardized hold and split biting task was used to assess the precision biting. The data was analyzed with non-parametric statistical tests. The results showed no significant differences in the hold forces, split forces, durations of split or peak split rates within or between the pain and pain-free conditions. The mean split rate increased significantly compared to baseline values both in the chronic patients and the pain-free condition. However, this increase was not evident in the experimental acute pain condition. Further, there were no significant differences in the mean split rates between the conditions. The data suggest that jaw muscle pain does not seem to alter precision biting in humans, however, the possibility that a nociceptive modulation of spindle afferent activity might have occurred but compensated for cannot be ruled out.

## Introduction

Chronic pain is a large and worldwide health problem with approximately 20% of the population reporting chronic pain of moderate to severe intensity (Breivik et al., 2006). The orofacial region is one of the most frequent locations for chronic pains, with a prevalence of 5–33% worldwide (Macfarlane et al., 2001, 2002, 2004; Isong et al., 2008; Schiffman et al., 2014). The most common diagnosis of the temporomandibular disorders (TMD) is masticatory muscle pain, i.e., myalgia (LeResche, 1997). Previous studies have shown that pain affects jaw motor control (Lund et al., 1991; Svensson and Graven-Nielsen, 2001; Murray and Peck, 2007) hence TMD is often accompanied by pain upon chewing, restricted mouth opening and impaired maximal (premolar/molar) bite force (Felício et al., 2002; Goiato et al., 2017; Xu et al., 2017). In the past, different theories have attempted to explain how pain and jaw function interrelate (Parker, 1990; Lund et al., 1991; Stohler, 1999; Svensson and Graven-Nielsen, 2001; Lobbezoo et al., 2006; Murray and Peck, 2007; Svensson and Kumar, 2016). However, several studies have either failed to or have presented contrary data to the fundaments of these theories (Stohler et al., 1988; Lund et al., 1991; Svensson et al., 1997, 1998; Svensson and Graven-Nielsen, 2001; Sae-Lee et al., 2008a, b). This contrasting evidence in the association between pain-related TMD and masticatory muscle function led to the integrated pain adaptation model which was presented in 2007 (Murray and Peck, 2007). Accordingly, pain is biopsychosocial and the relationship between pain and function may be more complex than proposed by the previous theories (Ohrbach et al., 2011, 2013; Bair et al., 2013). The theory proposes that just as an individual’s experience of pain varies, so will also an individual’s motor response.

Most evidence for how pain and jaw function interact comes from studies where pain was induced by injecting chemical substances in healthy subjects (Svensson and Graven-Nielsen, 2001; Graven-Nielsen, 2006; Kumar et al., 2015a, b). The use of pain models to mimic clinical pain is essential in research because the cause of pain is known and the effect can be controlled in such models. However, even if these pain models resemble clinical TMD-pain it is not certain that the induced pain can be exactly compared with the real, chronic TMD-condition. During a chronic pain condition changes in the nervous system occur causing central sensitization, which in turn may affect the motor function (Woolf and Walters, 1991; Woolf and Salter, 2000; Latremoliere and Woolf, 2009). Therefore, there is a greater need for studies that aim to investigate the mechanisms behind chronic musculoskeletal pain as well as further investigations of the contrasting evidence for the association between pain-related TMD and masticatory muscle function.

Human biting and chewing behaviors are controlled by a complex sensory-motor regulation involving the face primary motor cortex, the cortical masticatory area, and the central pattern generator (CPG) and coordinated by the sensory information from the periodontal mechanoreceptors (PMR’s), jaw muscle spindles, articular temporomandibular joint (TMJ) receptors, and pulpal mechanoreceptors as well as other orofacial features (Dellow and Lund, 1971; Nozaki et al., 1986; Lund, 1991; Trulsson and Johansson, 1996a, 2002; Türker, 2002; Lund and Kolta, 2006; Trulsson, 2006; van der Bilt et al., 2006; Grigoriadis et al., 2014; Kumar et al., 2014). Further, orofacial pain may be a potential modifier of mastication and jaw motor control (Clark et al., 1984) and therefore it is of great importance to investigate how pain affects human jaw function. Therefore, the aim of the study was to investigate the effect of chronic pain on oral motor force control during precision biting and compare it with experimentally induced acute pain in healthy controls. Our hypothesis was that chronic jaw muscle pain would affect the precise biting behavior and this would be reflected in the higher holding forces during the biting task (Capra et al., 2007), similar to the higher forces caused by alterations of the PMR’s (Svensson and Trulsson, 2011). Furthermore, we also hypothesized that the duration of the split phase should increase in chronic pain patients due to the fear of pain increase (Nicholas, 2007). On the other hand, the experimental acute pain model was not hypothesized to alter the fine motor control and would not affect the hold force and split duration in healthy participants similarly to other previously used models (Kumar et al., 2015a).

## Materials and Methods

This controlled, experimental, clinical study was conducted at the Department of Dental Medicine, Karolinska Institutet, Huddinge, Sweden. The study was performed in accordance with the declaration of Helsinki and approved by the Regional Ethical Review Board in Stockholm (DNR: 2014/1394-3). The chronic TMD-pain patients were recruited at the Specialist Clinic at the University Dental Clinic, Karolinska Institutet, Huddinge, Sweden and Eastmaninstitutet Folktandvården Stockholms län AB, Stockholm, Sweden. The healthy participants were recruited at the Department of Dental Medicine or the University Dental Clinic, Karolinska Institutet, Huddinge, Sweden. Before inclusion, all participants were given both verbal as well as written information about this study and an informed written consent was obtained.

### Participants

A sample size calculation revealed that 17 participants in each group was required to show a mean (SD) difference of 30% between the groups, with a power of 80% and a significance level of 0.05 (Svensson and Trulsson, 2011). Hence, 22 patients (mean age ± SD = 34 ± 12 years) with chronic myalgia including myofascial pain with referred pain in the masseter muscles with/without temporal myalgia were recruited in the study. Additionally, 22 pain-free, sex- and age-matched healthy volunteers (mean age ± SD = 33 ± 11 years), were also included in the study as controls. Therefore, the patient group comprised of 16 women (mean age ± SD = 35 ± 13 years) and six men (mean age ± SD = 30 ± 7 years), the control group also comprised of 16 women (mean age ± SD = 34 ± 13 years) and six men (mean age ± SD = 30 ± 6 years). The healthy controls also acted as the experimental acute pain group.

Further, out of the 22 included participants in each group 18 participants (14 women and 4 men) from the chronic patient group and 18 participants (12 women and 6 men) from the control group provided complete data for the analysis. The four remaining participants in each group had either incomplete data or were identified as outliers. This data was handled as missing data in the statistical analyses (identification of outliers is described further below).

The inclusion criteria for participation in the study were: (a) age over 18 years; (b) intact natural central incisors with normal relation to antagonistic teeth; (c) individuals capable to protrude the lower jaw in order to be able to perform the hold and split task. Exclusion criteria were: (1) a diagnosis of arthralgia, degenerative joint disease painful jaw clicking or locking according to (DC/TMD) (Schiffman et al., 2014); (2) clinically visible dental pathology or mobility, toothache, severe malocclusions, tooth wear grade 3 = exposure of pulp or secondary dentine according to the simplified scoring criteria for tooth wear index I (López-Frías et al., 2012), (3) earlier trauma to the anterior teeth; (4) root-canal treatments in the anterior teeth, orthodontic retainer, fixed prosthodontics (implants, bridges, crowns) in the anterior teeth, dentures; (5) systemic inflammatory diseases (i.e., rheumatoid arthritis, fibromyalgia, etc.), neuropathic pain or neurological disease; (6) whiplash associated disorder; (7) use of any medication that might influence the response of pain i.e., analgesics during 24 h preceding the experiment, use of cannabinoids, or any medication that might influence the neurological function; (8) allergy to any of the substances or food used in the experiment; (9) pregnancy or lactation; and (10) cognitive or physical disability that prevent participation.

Additional inclusion criteria *for patient group* were: (d) a diagnosis of local myalgia or myofascial pain or myofascial pain with referred pain in the masseter muscle according to the diagnostic criteria for temporomandibular disorders (DC/TMD); (e) a pain duration of at least 3 months; (f) current pain with a minimum score of 3 according to numeric rating scale (NRS 0-10).

*For the healthy individuals* the additional inclusion criteria were: (d) good general health. Additional exclusion criteria were: (11) a diagnosis of myalgia or myofascial pain according to the DC/TMD; (12) additional palpatory tenderness of the masseter, temporalis muscles or over the TMJ.

### Experimental Protocol

**FIGURE 1:**
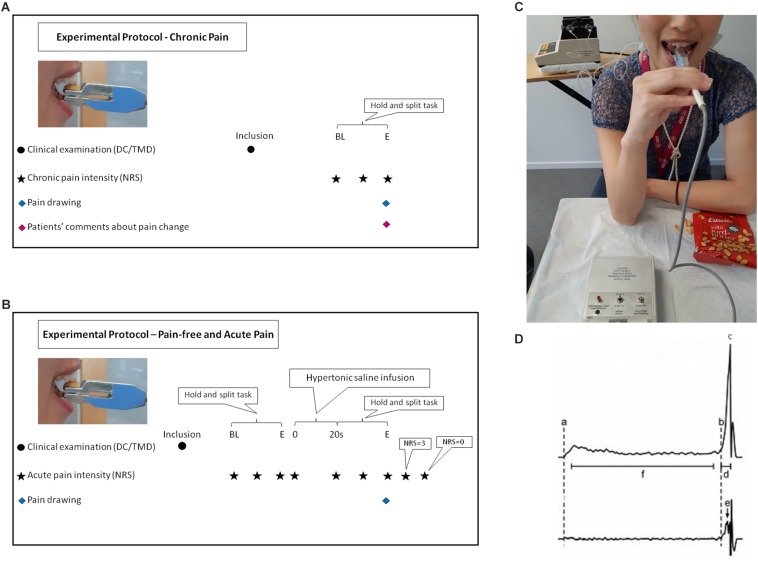
Flow-chart illustrates the experimental protocol for chronic pain **(A)**. BL, baseline before the task; BL, start of the task; E, end of the task. Flow-chart illustrates the experimental protocol for pain-free and experimental acute pain **(B)**. BL, baseline before the task; s, second; E, end of the task. DC/TMD, Diagnostic criteria for temporomandibular disorders; NRS, numeric rating scale. Participant performing the hold and split task and the equipment used during the experiment **(C)**. Estrella half peanut was placed on force transducer during acute induced pain in the masseter muscles bilaterally by infusion of hypertonic saline with Harvard infusion pump. A written informed consent was obtained from the participant in the figure for the publication of the image. A representative schematic force profile (upper trace) and force rate profile (lower trace) of one hold and split trial as shown in the WinZoom program **(D)**. (a) Initial contact with the food, (b) Initiation of splitting, (c) the split force and end of the split phase, (d) duration of the split phase, (e) peak rate of split force, and (f) hold phase, interval beginning 0.2 s after initial contact with the food and ending 0.2 s prior to the onset of the split phase.

Participants answered questionnaires regarding anxiety (generalized anxiety disorder scale-7; GAD-7) (Löwe et al., 2008), depression (the patient health questionnaire for depression-9; PHQ-9) (Kroenke et al., 2001), physical symptoms (PHQ-15, the patient health questionnaire for physical symptoms-15) (Kroenke et al., 2002), and stress (PSS-10, perceived stress scale-10) (Nordin and Nordin, 2013). Patients with myalgia answered questionnaires regarding chronic pain (graded chronic pain scale-7; G-7) (Von Korff et al., 1992). Prior to the inclusion, all participants were clinically examined according to DC/TMD. The experimental protocol and time points of assessments are illustrated in Figure 1A for the chronic pain and 1B for the pain-free and experimental acute pain. During the single experimental session of about 1 h, the chronic pain patients as well as the pain-free healthy controls were asked to perform ten trials of a standardized hold and split biting task (Trulsson and Johansson, 1996b) after performing five training trials. In order to simulate the experimental acute pain condition, simultaneous bilateral infusions of 0.4 ml of sterile hypertonic saline (58.5 mg/ml) into both masseter muscles were performed in the healthy controls during 20 s. The infusions were performed by an infusion pump (infusion rate 1200 μl/min; Harvard Infusion Pump 22, Harvard Apparatus, Great Britain) (Christidis et al., 2008), as shown in Figure 1C. The healthy controls were asked to perform ten trials while in pain. For all participants in all condition-groups, pain intensity was recorded before the task (for the experimental acute pain condition immediately after injection), at peak pain and at the end of the task. The experimental acute pain participants were also instructed to inform when the pain intensity was below the level of three on the 11-graded NRS-scale (0–10). While the chronic pain patients were asked to comment if their pain increased, decreased or remained unchanged after performing the hold and split task. After the task, all participants were asked to mark the maximum pain spread on a pain drawing. In the digital analysis, the scanned pain areas (i.e., the marks on the pain drawings) were analyzed in arbitrary units (au). Pain drawings provided visual illustration and quantitatively described the pattern and location of pain as well as referred pain (Wright, 2000). Three examiners (SA, AB, and KS) led the trials and were trained together in giving the instructions in the same manner according to a standardized protocol.

### Hold and Split Task

The hold and split task simulated the natural behavior of positioning/holding and contracting the jaw muscles to apply the optimum force needed for splitting food. The task was firstly described by Trulsson and Johansson in 1996 (Trulsson and Johansson, 1996b) using peanuts as test food. Many studies embraced the same methodology later on using a custom-built apparatus (Umeå University, Physiology Section, IMB, Umeå, Sweden) of the same design (Johnsen et al., 2007; Svensson and Trulsson, 2009, 2011; Kumar et al., 2014, 2015a, 2017, 2019). The apparatus consisted of 11 cm-long plastic-covered metal handle with a diameter of 7 mm connected to two duralumin blocks that terminate in two parallel rectangular plates. The total weight of the plates was 48 g and the stiffness between the plates was 50 N/mm while the total length of the apparatus was 17 cm. The upper duralumin block contained strain gauge force transducers for assessment of the forces applied to the plate (DC 200 Hz). The apparatus was designed to insure that the force assessment is independent of where the force was applied to the plate (Svensson and Trulsson, 2009). A half of a roasted and salted peanut (Estrella salta jordnötter; Estrella AB, Angered, Sweden) was placed on the free-end of the plate. A less than 0.1 mm thin piece of plastic-coated fabric tape on the top of the upper plate prevents the peanut from slipping while the apparatus was being positioned. The lower plate, which was placed with 8 mm distance between surfaces from the upper plate, was equipped with a piece of plexiglass designed to function as an anterior stop while positioning of the lower incisors. Participants used their preferred hand to place the apparatus between the upper and lower right or left central incisors in order to hold the apparatus in a horizontal position. The participants placed the lower plate on the lower incisor and slid the apparatus until the anterior stop was reached and the edge of the upper antagonist central incisor was positioned near the middle of the peanut, as shown in Figure 1C. The participants were instructed to hold the peanut between their incisors and not to use more force than necessary to control the peanut (Trulsson and Gunne, 1998). After approximately 3–5 s, the participants were asked to split the peanut. The forces applied by the incisors were continuously monitored during the task. If the peanut was dropped before the holding phase, a new trial with a new peanut was recorded. On the other hand, if the peanut was dropped before splitting, the trial was observed as a failure.

### Data Analyses

The force data were collected and analyzed using a customized software (WinSC/WinZoom v1.52.0.1; Umeå University, Umeå, Sweden) with 12-bit resolution at 800 Hz. Force rates were obtained by symmetrical numerical time differentiation ± 5 points from/to the force signal. The initial contact (a) and the onset of the split phase (b) were both reliably identified from the force-rate signal. The beginning of the split phase was detected at the point at which the force rate exceeded 5 N/s, the minimum rate of force increase that could be reliably detected in a single trial. The hold force was measured as the mean value of the force during the interval (f) – beginning 0.2 s after initial contact with the food (a) and ending 0.2 s before the onset (b) of the split phase. The split phase was characterized by a distinct rapid increase in force (b to c), which eventually split the food morsel. The split force/the end of the split phase (c) was defined as the peak force prior to the moment the morsel split, indicated by a rapid decrease in the force. The duration of the split phase (d) was defined as the time from the onset of the split phase (b) to the end of the split phase (c). The mean split force rate was defined as the force increase from the onset (b) to the end (c) of the split phase, divided by the duration of the split phase (d). The peak split force rate (e) defined as the max split force rate (steepest slope in the split phase profile) was identified by the WinZoom program. This is schematically shown in Figure 1D (Svensson and Trulsson, 2009).

Incomplete force trials as well as trials that were observed as failures due to dropping the peanut before splitting were handled as missing data. The peanut slippage was observed five times in the chronic condition (two patients), while this occurred once in the pain-free condition and once during acute pain condition (the same participant). Since the participants were not allowed to train more than five times before performing the hold and split task all trials’ profiles were manually checked and outliers were detected by using Adjusted Boxplot Method for skewed distributions (Brys et al., 2004, 2005; Hubert and Vandervieren, 2008). Outliers were handled as missing data. In all the three conditions (chronic pain, pain-free and experimental acute pain) only the first five “inlying” trials were included in the analyses (as for the pain conditions only trials during pain intensity of NRS ≥ 3). One participant from the healthy group did not answer the questionnaires hence; psychosocial data for that particular participant was handled as missing data.

The normality of the entire data was evaluated by using the Adjusted Boxplot Method and the Shapiro-Wilk test. The data showed a non-normal distribution with majority of the variables skewed to the right. Therefore, non-parametric tests were used to analyze the data. For each participant, data from all five included trials provided a participant median for each measurement and all data are further presented as group median (IQR, interquartile range). The data were analyzed with the SigmaStat software (version14.0; Systat Software Inc., San Jose, CA, United States) and for all tests, the level of significance was set at *P* < 0.05. Comparisons of psychosocial variables were done with Mann-Whitney Rank Sum test. For within condition’s comparisons, the non-parametric Friedman’s analysis of variance (ANOVA) for repeated measures and Tukey *post hoc* test for the associated multiple comparisons were used to test changes in all variables versus the baseline value of each variable (the first trial for each participant and condition was considered as baseline). For between conditions’ comparisons, Wilcoxon Signed Rank test and Mann–Whitney Rank Sum test were used. The variables included in the analyses were therefore conditions (chronic, pain-free, acute) and trials (Macfarlane et al., 2001, 2002; Breivik et al., 2006; Isong et al., 2008; Schiffman et al., 2014). For descriptive purposes, sex (men, women) was an additional variable included in the analyses. Spearman correlation test was applied to detect any correlation between force and pain variables.

## Results

### Psychosocial Characteristics

There were no significant differences between the chronic pain patients and the healthy participants regarding any of the psychosocial symptoms (Mann-Whitney Rank Sum test; *P* > 0.05) (Table 1).

**TABLE 1:** Pain and pyschosocial data.

		**All**			**Women**			**Men**	
**Chronic pain patients**									
Pain intensity (NRS)		3.50 (1.50)			3.50 (1.00)			3.50 (1.75)	
Peak pain intensity (NRS)		4.25 (3.50)			4.25 (3.50)			4.50 (3.50)	
Pain area (au)		584.84 (745.53)			537.89 (639.169)			851.90 (1354.763)	
Pain duration (months)		72.00 (85.50)			90.00 (78)			39.00 (25.50)	
**Experimental acute pain in healthy participants**									
Pain intensity after 20 s infusion (NRS)		7.00 (2.25)			6.50 (1.75)			7.50 (1.38)	
Peak pain intensity (NRS)		7.00 (1.625)			7.00 (2.625)			7.00 (1.625)	
Pain area (au)		195.99 (278.50)			185.75 (277.50)			323.73 (168.79)	
Pain duration to NRS = 3 s		216 (184.50)			244.00 (184.50)			202.00 (97.25)	

	**All patients**	**All healthy**	***P*-value**	**Female**	**Healthy**	***P*-value**	**Male**	**Healthy**	***P*-value**
		**participants**		**patients**	**women**		**patients**	**men**	

GAD-7^1^	22.22 (55.56)	16.67 (66.67)	1.00	16.67 (38.89)	11.11 (38.89)	0.70	5.56 (16.67)	5.56 (16.67)	1.00
PHQ-9^2^	19.44 (30.56)	8.33 (68.06)	0.34	16.67 (27.78)	5.56 (33.33)	0.49	5.56 (8.33)	2.78 (18.06)	0.89
PHQ-15^3^	27.78 (19.44)	16.67 (43.06)	0.69	22.22 (16.67)	13.89 (25)	0.89	5.56 (8.33)	2.78 (18.06)	0.89
PSS-10^4^	38.89 (16.67)	27.78 (22.22)	1.00	27.78 (16.67)	22.22 (22.22)	0.70	5.56 (5.56)	11.11 (5.56)	0.70

*Data are expressed as median (IQR, interquartile range). The forces were assessed in Newton (N), the duration in Seconds (s), and the rates in Newton per Seconds (N/s). *P*-values refer to the comparisons between trials by Friedman’s ANOVA and Tukey *post hoc* test. ^1^GAD-7: Generalized Anxiety Disorder (7 Questions). ^2^PHQ-9: The Patient Health Questionnaire for Depression (9 Questions). ^3^PHQ-15: The Patient Health Questionnaire for Physical Symptoms (15 Questions). ^4^PSS-10: Perceived Stress Scale Scoring (10 Questions).*

### Pain Characteristics

Majority of the chronic pain patients (61%) were diagnosed with local myalgia in the masseter muscles (with/without temporal myalgia) and 39% were diagnosed with myofascial pain with referred pain in the masseter muscles (with/without temporal myalgia) according to DC/TMD. 61% of the chronic patients had low pain intensity and low grade of disability (GCPS-7), 33% had high pain intensity and low grade of disability, about 6% had moderately limiting high disability and none of them had severely limiting high disability. The pain intensity, pain area and pain duration in the chronic patients are presented in Table 1.

The induced experimental acute pain intensity, pain area, and pain duration in the healthy participants are presented in Table 1.

### Force Characteristics

Healthy participants, both during pain-free and experimental acute pain condition, as well as chronic pain patients applied low forces during the hold phase followed by a rapid-ramp increase in force until the peanut split. There were no significant differences in the hold forces (pain-free: 0.99 N, acute pain: 1.46 N, chronic pain: 1.00 N) and the split forces (pain-free: 28.95 N, acute pain: 27.35 N, chronic pain: 30.79 N) within (Friedman/Tukey; *P* > 0.05) (Table 2) or between the conditions (Wilcoxon Signed Rank and Mann-Whitney Rank Sum; *P* > 0.05) (Table 3). Further, there were also no significant differences in the durations of split (pain-free: 0.26 s, acute pain: 0.30 s, chronic pain: 0.30 s) and the peak split force rates (pain-free: 307.38 N/s, acute pain: 285.28 N/s, chronic pain: 278.34 N/s) both within (Friedman/Tukey; *P* > 0.05) (Table 2) and between the conditions (Wilcoxon Signed Rank and Mann-Whitney Rank Sum; *P* > 0.05) (Table 3). The mean split force rate increased significantly compared to baseline values both in the chronic condition and the pain-free condition (Friedman/Tukey; *P* = 0.04 and 0.01, respectively) (Table 2). This increase in rate was not evident in the experimental acute pain condition (Friedman; *P* = 0.11) (Table 2). Observations of the mean split rates revealed no significant differences (pain-free: 99.76 N/s, acute pain: 82.07 N/s, chronic pain: 83.62 N/s) between the conditions (Wilcoxon Signed Rank and Mann-Whitney Rank Sum; *P* > 0.05) (Table 3).

**TABLE 2:** Changes compared to baseline values in force, duration and rate variables in the three conditions.

**Variable**	**Chronic**			**Pain-free**			**Experimental acute**		
			
	**Trial 1**	**Trial 5**	***P*-value**	**Trial 1**	**Trial 5**	***P*-value**	**Trial 1**	**Trial 5**	***P*-value**
Hold force (N)	0.84 (1.13)	1.09 (1.02)	0.85	0.99 (1.47)	1.14 (1.08)	0.43	1.29 (1.28)	1.76 (1.56)	0.12
Split force (N)	30.05 (14.09)	29.42 (7.71)	0.66	30.78 (14.94)	28.83 (7.72)	0.91	27.19 (14.49)	27.46 (14.78)	0.67
Duration of Split (s)	0.47 (0.56)	0.28 (0.38)	0.07	0.32 (0.34)	0.27 (0.28)	0.08	0.41 (0.53)	0.24 (0.20)	0.054
Split force increase (N)	24.69 (19.40)	27.00 (15.83)	0.92	29.50 (7.91)	26.12 (6.89)	0.46	24.40 (13.36)	22.89 (11.01)	0.51
Mean split rate (N/s)	63.62 (60.08)	69.59 (102.83)	0.04^∗^	85.23 (45.68)	128.83 (119.56)	0.01^∗^	59.56 (66.23)	85.99 (79.06)	0.11
Peak split rate (N/s)	226.89 (285.29)	370.36 (290.28)	0.15	226.89 (145.03)	396.22 (254.0)	0.06	266.93 (285.29)	238.36 (215.21)	0.37

*Data are expressed as median (IQR, interquartile range). The forces were assessed in Newton (N), the duration in Seconds (s), and the rates in Newton per Seconds (N/s). *P*-values refer to the comparisons between trials by Friedman’s ANOVA and Tukey *post hoc* test. ^∗^Significant difference *P* < 0.05.*

**TABLE 3:** Comparisons between the three conditions regarding force, duration, and rate variables.

**Variable**	**Chronic**	**Pain-free**	***P*-value**	**Experimental acute**	**Pain-free**	***P*-value**	**Chronic**	**Experimental acute**	***P*-value**
Hold force (N)	1.00 (1.30)	0.99 (1.13)	0.99	1.46 (0.94)	0.99 (1.13)	0.06	1.00 (1.30)	1.46 (0.94)	0.28
Split force (N)	30.79 (5.28)	28.95 (5.62)	0.19	27.35 (7.50)	28.95 (5.62)	0.55	30.79 (5.28)	27.35 (7.50)	0.07
Duration of split (s)	0.30 (0.22)	0.26 (0.21)	0.32	0.30 (0.20)	0.26 (0.21)	0.33	0.30 (0.22)	0.30 (0.20)	0.99
Split force increase (N)	27.70 (11.01)	28.09 (5.09)	0.83	23.43 (7.32)	28.09 (5.09)	0.01^∗^	27.70 (11.01)	23.43 (7.32)	0.12
Mean split rate (N/s)	83.62 (80.63)	99.76 (57.61)	0.28	82.07 (55.51)	99.76 (57.61)	0.12	83.62 (80.63)	82.07 (55.51)	0.84
Peak split rate (N/s)	278.34 (240.24)	307.38 (228.56)	0.40	285.28 (142.84)	307.38 (228.56)	0.10	278.34 (240.24)	285.28 (142.84)	0.94

*Data are expressed as median (IQR, interquartile range). The forces were assessed in Newton (N), the duration in Seconds (s), and the rates in Newton per Seconds (N/s). *P*-values refer to the comparisons between conditions by Wilcoxon Signed Rank and Mann-Whitney Rank Sum tests. ^∗^Significant difference *P* < 0.05.*

### Pain Intensity in Relation to Force Variables

The worst reported pain intensity during trial (NRS = 4.25) in the chronic condition correlated negatively with the hold force (1.00 N) (Spearman correlation; *P* = 0.04). The pain intensity 20s after infusion (NRS = 7.00), and the worst reported pain intensity during trial (NRS = 7.00) showed a negative correlation with the duration of the split phase in the experimental acute pain condition (0.30 s) (Spearman correlation; *P* < 0.05).

## Discussion

The findings of the present study showed that neither experimental acute pain nor chronic muscular pain affects the human jaw motor control during a standardized precision biting task involving “holding” and “splitting” of peanuts with anterior teeth. The findings seem to indicate that jaw muscle pain does not alter the sensorimotor regulation and precision control of the jaws.

The PMR’s are innervated by fibers terminating in subnucleus interpolaris. The signaling in these fibers is faster and probably not affected by noxious stimuli from trigeminal facial areas that terminate more caudally in subnucleus caudalis. The rostral projections are larger in diameter and thereby faster than the more caudally located ones (Capra and Dessem, 1992). Further, the muscle spindles are innervated by efferent nerve fibers, motorneurons, which receive information from the central nervous system (CNS). The motorneurons convey nervous impulses to produce muscular effect, causing muscle fibers to shorten and contract. To enhance contraction either the firing frequency of each neuron increases or more motor units are activated/recruited (McComas, 1998; Loeb and Ghez, 2000; Miles, 2004). The fine-tuning of this activity is achieved by sensory information from orofacial receptors including the PMR’s and muscle spindles to the CNS. The results seem to indicate that the presence of healthy PMR’s and pulpal receptors provide the CNS with accurate sensory information despite the muscular pain. There is a possibility that a nociceptive modulation of spindle afferent activity might have occurred (Capra et al., 2007) but compensated for, hence resulting in undetectable effect on the force parameters in the present study. Activity along slower conducting nociceptive afferents could still modify the activity of faster afferents via intra-nuclear connections within the trigeminal brainstem sensory nuclear complex. Some masseter nociceptive afferents might provide axon collaterals to the rostral trigeminal subnuclei (Capra and Dessem, 1992). Nevertheless, this study did not assess nociceptive afferent activity. Furthermore, a potential effect of the experimentally induced pain (Nash et al., 2010; Minami et al., 2013) might have been compensated by other unaffected muscle parts/muscles. This assumption could be applicable on the chronic pain condition as well. The chronic pain patients may over time have developed compensating motor and behavioral strategies (Mohn et al., 2011; Fillingim et al., 2018).

### Hold Phase

The fact that all the conditions showed no significant differences in the hold forces compared to the baseline trials indicated no trial order effect within the conditions. This is in line with results from previous studies where the hold and split task was used (Kumar et al., 2014; Zhang et al., 2016). The hold force in the pain-free condition was found to be 0.99 N in this study and in line with the previously mentioned study (Kumar et al., 2014) as well as earlier reports where the hold force for peanuts in healthy participants with natural dentition during normal conditions varied between 0.59 and 0.79 N (Trulsson and Johansson, 1996b; Trulsson and Gunne, 1998; Johnsen et al., 2007; Svensson and Trulsson, 2009, 2011).

The chronic pain patients applied similar hold force as the pain-free participants. During the holding phase in pain-free healthy individuals, it has been hypothesized that the PMR’s besides the afferent nerve fibers in muscle spindles play a role in signaling the early contact-state information about the peanut in a predictive feed-forward manner in order to activate the motor commands that are needed for initiating the split phase (Svensson and Trulsson, 2009, 2011). The jaw muscle activity increases in response to an increased food hardness. It was shown that the destruction of either periodontal or muscle afferents in animals, reduced such increase in jaw muscle activity (Lavigne et al., 1987; Morimoto et al., 1989; Hidaka et al., 1997). However, chronic muscle pain did not seem to have any effect on the forces during the holding phase in this study. One probable explanation could be that the presence of healthy PMRs are of greater importance in oral fine motor control than the muscle spindles (Kumar et al., 2019).

### Split Phase

The split phase was characterized by a sudden rapid ramp increase in the bite force. When the peanut was split, the force dropped down and an unloading of the teeth occurred (Svensson and Trulsson, 2009, 2011). The unloading contributes to a reflex response in the masseter muscle activity that results in stopping the jaw closing movement (Turker and Jenkins, 2000). All conditions showed no differences in the split force and duration of split phase compared to baseline values. This finding is in line with previous studies where experimental acute pain did not show any robust effects on the split forces (Kumar et al., 2014, 2015a). It was previously suggested that split forces and duration of split are mainly dependent on the mechanical properties of the food and the bevel of the incisal edges during biting with anterior teeth (Johnsen et al., 2007; Svensson and Trulsson, 2009, 2011; Kumar et al., 2014, 2015c, 2017). In healthy individuals in previous studies, the split forces varied between 17.6 and 35.9 N and the duration of the split phase varied between 0.22 and 0.34 s (Trulsson and Johansson, 1996b; Svensson and Trulsson, 2009, 2011). In our study the split force was 28.95 N and the duration of the split phase was 0.26 s in healthy pain-free participants. Our findings were within the range of the previous results.

The significant increase in the mean split force rate in the pain-free individuals and chronic pain patients indicates that reaching to the split of the peanut from the initiation of the split phase was faster compared to baseline. On the other hand, no statistically significant difference in the mean split force rate in the acute condition compared to baseline may indicate that the high intensity of the induced pain and the protective effect of the acute pain affected the rate. However, the differences were only found within the conditions (not between them) and there were no differences in the duration of the split (one of the two variables that the mean split force rate depends on) in all the three conditions. Further statistical analyses showed that there was a significant difference in the split force increase (the other variable that the mean split force rate depends on) between the acute and the pain-free conditions where the acute condition showed a smaller increase (Table 3). This significant difference could not be found between the chronic and the pain-free conditions or between the chronic and acute conditions. Moreover, no significant differences could be found within the three groups in the split force increase (Table 2). Therefore, this finding should be considered with caution and further investigations would be needed in order to make a more conclusive interpretation. It was suggested that the individual’s reaction to pain might depend on the specifics of the performed task (Sae-Lee et al., 2008a, b). An individual’s confidence that they can manage pain (self-efficacy beliefs) predicts avoidance behavior (Nicholas, 2007) and the ability to persist with a task (Turner et al., 2005). The smaller split force increase in the acute condition could be an indication that the participants could predict the split force needed easier than the two other condition groups. The acute condition group did not need to increase the mean split force rate as much as the other two condition groups since the split force onset was already high and more approximate to the split force needed. That is in line with the significant increase in the mean split force rate in the pain-free and chronic conditions. A possible explanation could be a training effect since the participants in the acute condition were the same persons as in the pain-free condition. However, the previously cited studies (Kumar et al., 2014, 2015a; Zhang et al., 2016) did not report any findings about rate variables. There were no significant differences in the mean and peak split force rates between the three conditions indicating that the pain did not impair the neural control needed for achieving the necessary force magnitude needed for splitting the peanut.

For the acute jaw muscle pain, the results confirms our hypothesis and are in line with previous studies using other experimental jaw muscle pain models and in other populations which had shown that experimental acute pain had no detectable or robust effect on the hold and split forces and on the variability of the forces, duration of the split phase, electromyographic muscle activity (EMG) or jaw movement amplitude in comparison with healthy controls during biting and mastication (Svensson et al., 1997; Kumar et al., 2015a, b) indicating the absence of motor impairment even in subjects who reported moderate to intense levels of pain. Interestingly, the results showed that this was the case even for the chronic jaw muscle pain which was contradictory to our hypothesis. On the other hand, this is in line with results from a previous study (Goiato et al., 2017) showing that maximal bite forces in the incisor region was similar prior to and after jaw muscle pain relief, suggesting that the presence of pain did not affect the maximal forces in this region.

### Study Limitations and Strengths

The baseline pain intensity in chronic pain patients was rather low, none of them had a severely limiting high disability according to GCPS-7 and pain catastrophizing according to pain catastrophizing scale-13 (PCS-13) (Boonstra et al., 2016) was not assessed in this study which could be considered as a limitation. The fact that there were no significant differences between chronic patients and healthy participants regarding the psychosocial characteristics minimized the possible confounding effect, and therefore could be considered a strength of the current study.

Incisors were used in this study in order to minimize confounding factors as it had earlier been reported that participants felt it was easier to master the task when anterior teeth were used compared to posterior teeth (Johnsen et al., 2007). It had been also shown that the masseter muscle was significantly more active (higher EMG) than the temporal muscle during tasks involving incisal biting and jaw protrusion (Farella et al., 2008) and that biting in a protrusive position was accompanied by the highest activation of the masseter muscle (Lu et al., 2013). One can assume that there would be a practicing bias in the experimental acute pain condition since the same individuals performed the task twice, pain-free and while in experimental acute pain. However, the circumstances differed and a previous study showed that there was no apparent or optimization of jaw motor control when this specific task was repeated up to sixty times, in participants with healthy periodontium (Kumar et al., 2014).

### Conclusion

The current study shows that jaw muscle pain does not seem to alter precision biting in humans. Specifically, chronic myalgia in the jaw muscles as well as experimentally induced acute pain did not show any effects on the hold or split forces when compared to healthy pain-free individuals. However, a possibility that a nociceptive modulation of spindle afferent activity might have occurred but compensated for cannot completely be ruled out.

## Data Availability Statement

The datasets for this study will be made available by the authors, without undue reservations, to any qualified researcher.

## Ethics Statement

The studies involving human participants were reviewed and approved by the Regional Ethical Review Board in Stockholm (DNR: 2014/1394-3). The patients/participants provided their written informed consent to participate in this study.

## Author Contributions

SA wrote the manuscript, performed the research, analyzed the data, and made the tables and figures. AB performed the research and participated in manuscript editing. KS participated in the design of the project, performed the research, and participated in manuscript editing. AK participated in data analyses and manuscript editing. AG designed the project and participated in manuscript editing. NC designed the project, and participated in data analyses, making the figures, and manuscript editing.

## Conflict of Interest

The authors declare that the research was conducted in the absence of any commercial or financial relationships that could be construed as a potential conflict of interest.

## Abbreviations

ANOVA: analysis of variance
au: arbitrary units
cm: centimeter
CNS: central nervous system
CPG: central pattern generator
DC-TMD: diagnostic criteria for temporomandibular disorders
EMG: electromyographic muscle activity
G: gram
GAD-7: generalized anxiety disorder scale-7
GCP-7: graded chronic pain scale-7
Hz: hertz
IQR: interquartile range
mg: milligram
min: minute
ml: milliliter
mm: millimeter
N: Newton
NRS: numeric rating scale
PHQ-15: the patient health questionnaire for physical symptoms-15
PHQ-9: the patient health questionnaire for depression-9
PMR: periodontal mechanoreceptor
PSS-10: perceived stress scale-10
s: second
SCON: Scandinavian Center for Orofacial Neurosciences
SD: standard deviation
TMD: temporomandibular disorders
TMJ: temporomandibular joint
US: United States
USD: United States dollar
μ l: microliter.
